# Depression, Anxiety, Stress and Hyperemesis Gravidarum: Temporal and Case Controlled Correlates

**DOI:** 10.1371/journal.pone.0092036

**Published:** 2014-03-17

**Authors:** Peng Chiong Tan, Syeda Nureena Zaidi, Noor Azmi, Siti Zawiah Omar, Su Yen Khong

**Affiliations:** Department of Obstetrics and Gynaecology, University of Malaya, Lembah Pantai, Kuala Lumpur, Malaysia; VU University Medical Center, Netherlands

## Abstract

**Objective:**

To evaluate the temporal and case-controlled correlations of anxiety, depression and stress with hyperemesis gravidarum

**Study Design:**

We performed a longitudinal cohort study of women with hyperemesis gravidarum using the Depression, Anxiety and Stress Scale (DASS-21) to evaluate psychological distress at hospitalization and in the third trimester of pregnancy (from 28 weeks gestation). Third pregnancy trimester controls were recruited from routine antenatal clinic attendees who were matched to gestational age at the second DASS-21 assessment in the HG cohort.

**Results:**

The prevalences of nausea and vomiting, depression, anxiety and stress caseness in newly hospitalised hyperemesis gravidarum women were 100% and 100%, 19%, 69% and 21% which by the third trimester had fallen to 15.7% and 9.9%, 4%, 19% and 3% and in third trimester controls were 15.9% and 14.2%, 14%, 61% and 20% respectively. Within the hyperemesis gravidarum cohort, nausea, vomiting depression, anxiety and stress reduced significantly by an absolute 84.3% (95% CI 76.2%–89.8%), 90.1% (82.8%–94.2%), 14.9% (7.2%–23.0%), 49.6% (38.6%–58.7%) and 18.2% (10.4%–26.4%) respectively between hospitalization for hyperemesis gravidarum and at the third trimester. In the third trimester, when comparing the hyperemesis gravidarum cohort to controls, the risk of nausea or vomiting was similar but depression, anxiety and stress were significantly lower: adjusted odds ratio AOR 0.10 (95% CI 0.03–0.5), 0.11 (0.05–0.23) and 0.08 (0.02–0.33) respectively.

**Conclusion:**

Our study revealed a reassuring pattern of a strong rebound from depression, anxiety and stress in women with hyperemesis gravidarum such that by the third pregnancy trimester the level of psychological distress was even lower than in controls. This observation imply that much of the psychological distress in acute hyperemesis gravidarum is self-limiting and probably in the causal pathway of hyperemesis gravidarum. Care in women with hyperemesis gravidarum should focus on the relief of nausea and vomiting.

## Introduction

Hyperemesis gravidarum (HG) complicates around 0.3–2% of pregnancies and is characterized by severe nausea and vomiting due to pregnancy resulting in dehydration and electrolyte imbalance, metabolic disturbance and the need for hospitalization.[1] HG typically arises between the 4th and the 10th week of gestation with resolution usually by 20 weeks of gestation[2] but in 10% of cases symptom may persist throughout pregnancy[3].

Hormonal relationships with HG are often reported particularly the association with high levels of human chorionic gonadotropin. The aetiology of HG remains unclear and may be multi-factorial with biologic, psychological and socioeconomic antecedents.[2] Historically, a pregnant woman's vomiting was thought to represent various psychological conflicts but it is also plausible that psychological symptoms are a result of the stress and the physical burden of HG rather than a cause.[2] Women with prior psychiatric or medical conditions are more likely to develop HG when pregnant.[4] The prevalence of major depression, generalized anxiety disorder, avoidant personality disorder and obsessive-compulsive personality disorder has been shown to be higher in women with HG.[5] In contrast, women with HG were no more likely than controls to have psychological morbidity after birth.[6]

A study at our centre indicates that anxiety and depression were common in HG women when assessed at their first hospitalization with caseness rates of 46.9% and 47.8% respectively.[7] These rates compare unfavourably with anxiety and depression rates of 36.3% and 22.1% in the first pregnancy trimester, 32.3% and 18.9% in the second trimester and 35.8% and 21.6% in the third trimester from a longitudinal study of Chinese women in Hong Kong.[8]

We sought to evaluate the evolution of nausea, vomiting, depression, anxiety and stress in the HG cohort from hospitalization into the third trimester of pregnancy. We hypothesize that psychological distress is a consequence of the symptom burden of HG. We expect that in the third pregnancy trimester the rates of anxiety, depression and stress caseness should fall back to the background rate or lower as HG symptoms resolved according to its natural history[9] whereas the natural history of depression and anxiety caseness rates during pregnancy is a small dip in the midtrimester followed by a rise in late pregnancy[8].

## Materials and Methods

Ethical approval for the study was provided by our institutional review board, the University of Malaya Medical Centre Medical Ethics Committee (reference number 806.9 dated 23 August 2010). The study recruited from 8 September 2010 to 17 February 2012. The study was conducted in University of Malaya Medical Centre a full service state funded university hospital in a city setting providing free or subsidized health care to the general public. Women hospitalised for hyperemesis gravidarum were identified on the ward as soon as possible after admission and approached to participate. The patient information sheet was provided and the recruiting provider handled queries as presented. Participants gave their written consent.

### Selection criteria

Inclusion criteria for participation were first hospitalization in the current pregnancy for the treatment of HG and ultrasound confirmation of pregnancy (or a positive urine pregnancy if an ultrasound was uninformative due to very early pregnancy). Women were excluded if they had multiple pregnancies, thyroid disease, gestational trophoblastic disease, established psychiatric illness or any other acute illness that could cause nausea and vomiting which may confound the diagnosis of HG.

### DASS-21

We used the Depression, Anxiety and Stress Scales (DASS) to assess the aforementioned types of psychological distress at hospitalization for HG and again in the third pregnancy trimester (from 28 weeks gestation). DASS was originally formulated as a 42 stem questionnaire[10]; a short form 21 stem version (DASS-21) can also validly be used to measure the dimensions of depression, anxiety, and stress in the general adult UK population[11]. There is a validated Malay language version of DASS-21[12]; the Malay language version has concurrent validity in anxiety and depression components in infertility patients[13] when compared to the Hospital Anxiety and Depression Scale which is validated in perinatal populations[14]. DASS-21 has 21 stems with 4 gradated answer choices to each stem. Each answer is scored 0 to 3. It comprises of 3 subscales for depression, anxiety and stress - each with 7 stems. The subscale scores were summated, and then doubled in value to bring the scoring in line with the original 42-stem DASS: threshold values for depression, anxiety and stress caseness were set at ≥10, ≥ 8 and ≥ 15 respectively.[15] The concept of caseness is introduced rather than a definitive case as DASS-21 is not a diagnostic test.

The DASS-21 questionnaire and data collection sheet for personal and medical characteristics were given to participants for completion. Instructions for form completion were provided by providers. The forms were collected after completion and checked. If the questionnaire or data sheet was incomplete, another request was made for the missing information but if a deliberate choice was made to not provide specific information, this was fully respected. The DASS-21 questionnaire was scored. If there was concern arising from participants' response, a discussion with the participant to ascertain appropriateness for formal psychiatric evaluation was made. Study data was not routinely made available to providers.

### Data collection

Inpatient data from the HG hospitalization were transcribed to the case report form from clinical notes and on-line laboratory records. Ketonuria, hyponatraemia, hypokalaemia and high haematocrit on admission and prolonged hospital stay were selected as measures of severity of HG at hospitalization. Standard inpatient care of HG was provided to participants: comprising initial rehydration with intravenous fluids (typically normal saline), an intravenous anti-emetic (typically metoclopramide) and oral thiamine. Oral intake was resumed when tolerated. Patients were discharged once they were rehydrated, electrolyte repleted and tolerating sufficient oral intake. Participants who wished to deliver in our centre were referred to the antenatal care clinic for subsequent care. Our pregnancy care system is open to local women of all risk categories who choose our services on their own volition.

Participants' clinical files were tagged to allow identification in the antenatal clinic for follow-up DASS-21 assessment and to provide information on nausea and vomiting within the last week. Follow up DASS-21 assessment was scheduled for as soon as possible after 28 weeks gestation when participants attended for routine care in our antenatal clinic. We selected 28 weeks as the threshold gestational age for reassessing nausea, vomiting, depression, anxiety and stress as it represents the conventional cut-off for the third pregnancy trimester, HG typically would have resolved by 20 weeks[2] allowing two or more months for psychological distress to respond and to minimize dropouts due to preterm delivery.

Participants who did not continue with antenatal care in our centre were identified through a search of antenatal clinic records and they were contacted through all available communication channels to arrange a mutually convenient appointment on or after 28 weeks of gestation for DASS-21 assessment in our gynaecology or antenatal clinic. DASS-21 may also be dispatched to be completed if requested. At least two attempts were made to obtain the follow up DASS-21 assessment.

### Controls

Controls matched for gestational age ≥ 28 weeks (at timing of second DASS-21 assessment in the HG cohort) were recruited on a 1 to 1 ratio from amongst routine antenatal clinic attendees by a co-author (SNZ). The inclusion criteria for the controls were the same as HG cases i.e. women with multiple pregnancies, thyroid disease or overt history of psychological illness were excluded. We could not recruit controls from the early first trimester at a similar gestational age to the HG cohort on their hospitalization as in our care set up women with normal pregnancies do not present to our hospital for routine care that early. Controls were recruited opportunistically on a first available basis from the antenatal clinic queue pool whilst waiting to be seen. Controls provided similar personal information using identical documents (including DASS-21) omitting information specific to hospitalization for HG. Controls that had probable HG (i.e. severe nausea and vomiting requiring medical treatment) in the current pregnancy were excluded.

### Sample size calculation

Study population sample size for the HG cohort, comparing depression caseness in early and late pregnancy was calculated thus. Depression caseness was found in 47.8% at hospitalization for HG[7] and present in 21.6% of the unselected third trimester antenatal population[8]. In about 10% of cases, symptoms of HG can persist into the third trimester.[3] Hence assuming that depression caseness rate approximates to that of the unselected antenatal population in recovered HG cases (21.6%) but remained at the initial rate (47.8%) in the 10% with continuing HG symptoms, the depression caseness rate is calculated to be 24.2% in the HG cohort in the third trimester. Applying alpha of 0.05, power 80%, prior depression caseness rate of 47.8% and third trimester caseness rate of 24.2%, using McNemar's test, 100 women are needed for a suitably powered study (online calculator via http://www.statstodo.com/SSizMcNemar_Pgm.php#Single calculation: sample size estimation). 41% of women hospitalised with HG to our centre did not go on to receive antenatal care and deliver at our centre.[1] Assuming a dropout rate of 40% in participants who did not deliver with us and a 10% dropout rate in those who delivered at our centre, 129 women with HG needed to be enrolled.

### Statistical Analysis

Statistical analysis was performed using the SPSS 15 (SPSS Inc., Chicago IL, USA). The one sample Kolmogorov-Smirnoff test was used to assess normality of data distribution. Fisher's exact test was used for 2×2 categorical data, Chi-Square test for larger than 2×2 categorical data sets. The means of normally distributed continuous data was assessed by Student t-test. The Mann Whitney U test was used for non-normally distributed data and ordinal data. McNemar's test was used to analyse change in nausea, vomiting, depression, anxiety and stress at hospitalization for HG compared to at the third trimester. Multivariable logistic regression analysis was used to control for co-variables with P<0.05 on bivariate analysis. P<0.05 on 2-sided tests was taken as a level of significance for all tests.

## Results

129 women were recruited at hospitalization for HG. All completed the DASS-21 questionnaire and also provided personal information as requested in our standardised data collection form. Eight participants (five could not be contacted or did not respond and three had miscarried) did not complete the DASS-21 questionnaire in the third trimester leaving 121 women in the HG cohort for analysis. 120 controls in their third trimester matched for gestational age (≥ 28 weeks gestation) were recruited from amongst regular antenatal clinic attendees. They also completed the DASS-21 questionnaire and provided the same personal data. Seven of the controls reported a history of probable HG with severe nausea and vomiting requiring medical treatment earlier in their pregnancy and were excluded, leaving 113 controls for analysis. We approached participants with worrying DASS-21 scores for discussion with an offer for a formal psychiatric appointment as appropriate. The offer was not taken up.

Table 1 shows the characteristics of the HG cohort at hospitalization stratified according to their depression, anxiety and stress caseness or non-caseness status to identify potential predictors using bivariate analysis. The rates of depression, anxiety and stress caseness were 19%, 69% and 21% respectively. We adjusted for all characteristics with P<0.05 on bivariate analysis in a multivariable logistic regression analysis model to identify independent risk factors for depression, anxiety and stress caseness. No significant independent predictor for depression, anxiety and stress was found after adjustment.

**Table 1:** Characteristics of women with hyperemesis gravidarum at hospitalization stratified according to their depression, anxiety and stress status as assessed by the 21-stem Depression Anxiety and Stress Scales.

	Depression*		P Value	Adjusted P‡	Anxiety†		P Value	Adjusted P‡	Stress§		P Value
	Yes n = 23	No n = 98			Yes n = 83	No n = 38			Yes n = 26	No n = 95	
Age (years)	28.4±4.5	28.8±4.8	P = 0.67		28.9±4.8	28.5±4.5	P = 0.69		29.5±4.6	28.6±4.7	P = 0.39
Gestational age (weeks)	9.4±3.4	9.3±2.2	P = 0.87		9.0±2.5	10.1±2.4	P = 0.03	P = 0.94	9.3±2.8	9.4±2.4	P = 0.99
Weight (kg)	55.5±11.4	55.5±10.7	P = 0.98		55.9±10.3	54.6±11.9	P = 0.53		54.5±11.2	55.8±10.7	P = 0.60
Gestational age at start of vomiting (weeks)	7.2±3.1	7.1±1.9	P = 0.85		6.8±2.1	7.9±2.2	P = 0.01	P = 0.20	6.8±2.6	7.2±2.0	P = 0.36
Duration of vomiting (weeks)	2.2±1.4	2.2±1.3	P = 0.94		2.2±1.3	2.2±1.4	P = 0.58		2.5±1.4	2.1±1.3	P = 0.16
Vomiting episodes (per day)	8 [5]–[10]	6.5 [5]–[10]	P = 0.15		8 [5]–[10]	6 [5–8.25]	P = 0.15		8 [5]–[10]	7 [5]–[10]	P = 0.15
Parity	1 [0–1]	0 [0–1]	P = 0.55		1 [0–1]	0 [0–2]	P = 0.55		1 [0–2]	0 [0–1]	P = 0.55
Miscarriage	4 (17.4)	17 (17.3)	P = 0.99		14 (16.9)	7 (18.4)	P = 0.80		4 (15.4)	17 (17.9)	P = 0.99
Ethnicity			P = 0.40				P = 0.18				P = 0.61
*Malay*	17 (73.9)	79 (80.6)			68 (81.9)	28 (73.7)			20 (76.9)	78 (80.0)	
*Indian*	3 (13.0)	10 (10.2)			10 (12.0)	3 (7.9)			4 (15.4)	9 (9.5)	
*Chinese*	2 (8.7)	2 (2.0)			2 (2.4)	2 (5.3)			0 (0.0)	4 (4.2)	
*Others*	1 (4.3)	7 (7.1)			3 (3.6)	5 (13.2)			2 (7.7)	6 (6.3)	
Previous hyperemesis gravidarum	6 (26.1)	25 (25.5)	P = 0.99		20 (24.1)	11 (28.9)	P = 0.66		9 (34.6)	22 (23.2)	P = 0.31
Planned pregnancy	12 (52.2)	52 (53.1)	P = 0.99		42 (50.6)	22 (57.9)	P = 0.56		11 (42.3)	53 (55.8)	P = 0.27
Married	21 (91.3)	97 (99.0)	P = 0.09		80 (96.4)	38 (100)	P = 0.55		26 (100)	92 (96.8)	P = 0.99
Local family support	20 (87.0)	79 (80.6)	P = 0.57		67 (80.7)	32 (84.2)	P = 0.80		23 (88.5)	76 (80.0)	P = 0.40
Low income∥	9 (39.1)	44 (44.9)	P = 0.65		36 (43.4)	17 (44.7)	P = 0.99		7 (26.9)	46 (48.4)	P = 0.07
Housing			P = 0.01	P = 0.06			P = 0.72				P = 0.48
*Owned*	6 (26.1)	39 (39.8)			29 (34.9)	16 (42.1)			11 (42.3)	34 (35.8)	
*Rented*	12 (52.2)	55 (56.1)			48 (57.8)	19 (50.0)			12 (46.2)	55 (57.9)	
*Living with extended family*	5 (21.7)	4 (4.1)			6 (7.2)	3 (7.9)			3 (11.5)	6 (6.3)	
Tertiary Education	10 (43.5)	60 (61.2)	P = 0.16		49 (59.0)	21 (55.3)	P = 0.70		15 (57.7)	55 (57.9)	P = 0.99
Paid employment	20 (87.0)	83 (84.7)	P = 0.99		71 (85.5)	32 (84.2)	P = 0.99		22 (84.6)	81 (85.3)	P = 0.99
Regular exercise	2 (8.7)	22 (22.4)	P = 0.24		15 (18.1)	9 (23.7)	P = 0.47		2 (7.7)	22 (23.2)	P = 0.10
Ketonuria			P = 0.42				P = 0.75				P = 0.57
*Nil*	1 (4.3)	3 (3.1)			2 (2.4)	2 (5.3)			1 (3.8)	3 (3.2)	
*1+*	0 (0)	4 (4.1)			2 (2.4)	2 (5.3)			0 (0)	4 (4.2)	
*2+*	0 (0.0)	1 (1.0)			1 (1.2)	0 (0.0)			0 (0)	1 (1.1)	
*3+*	7 (30.4)	15 (15.3)			16 (19.3)	6 (15.8)			7 (26.9)	15 (15.8)	
*4+*	15 (65.2)	75 (76.5)			62 (74.7)	28 (73.7)			18 (69.2)	72 (75.8)	
Hyponatraemia¶	15 (65.2)	76 (77.6)	P = 0.28		61 (73.5)	30 (78.9)	P = 0.65		20 (76.9)	71 (74.7)	P = 0.99
Hypokalaemia#	0 (0.0)	16 (16.3)	P = 0.04	P = 0.99	10 (12.0)	6 (15.8)	P = 0.57		3 (11.5)	13 (13.7)	P = 0.99
Long Hospital stay ≥ 4 days**	4 (17.4)	18 (18.4)	P = 0.99		17 (20.5)	5 (13.2)	P = 0.44		4 (15.4)	18 (18.9)	P = 0.78
High Haematocrit ≥ 0.41**	5 (22.7)	20 (20.6)	P = 0.78		19 (23.2)	6 (16.2)	P = 0.47		4 (15.4)	21 (22.6)	P = 0.59

Data presented as mean ± standard deviation, median [interquartile range] and number (%). Bivariate analyses are with Student t test for continuous data set, Mann Whitney U test for ordinal or non-parametric data, Fisher Exact test for 2×2 categorical dataset and Chi Square test for larger categorical dataset. All tests are 2-sided. Multivariable logistic regression analysis performed if multiple co-variables with bivariate P<0.05 found, incorporating in the model all the significant co-variables to identify independent risk factors for depression, anxiety and stress respectively.

*A calculated score of at least 10 on the summated (then doubled) scores of the depression component of the 21-stem Depression, Anxiety and Stress Scales

† A calculated score of at least 8 on the summated (then doubled) scores of the anxiety component of the 21-stem Depression, Anxiety and Stress Scales

‡ Multivariable logistic regression performed incorporating co-variables with P<0.05 on bivariate analyses where available to obtain adjusted P value.

§ A calculated score of at least 15 on the summated (then doubled) scores of the stress component of the 21-stem Depression, Anxiety and Stress Scales

∥ Month income of less than RM3000 (approximately US$950)

¶ Serum sodium level ≤ 135 mmol/L as defined by the normal range provided by our laboratory

# Serum potassium level ≤ 3.5 mmol/L as defined by the normal range provided by our laboratory

**Cut-offs defined as top quartile values for these parameters in the HG cohort

Table 2 shows the magnitude of the evolution of symptoms of nausea, vomiting, depression, anxiety and stress over time from hospitalization to the third trimester (on or after 28 weeks gestation) in the HG cohort. Nausea, vomiting, depression, anxiety and stress caseness all declined significantly (P<0.001, McNemar's test) as anticipated: absolute percentage reductions by the third trimester were 84.3% (95% CI 76.2–89.8%), 90.1% (95% CI 82.8–94.2%), 14.9% (95% CI 7.2–23.0%), 49.6% (95% CI 38.6–58.7%) and 18.2% (95% CI 10.4–26.4%) respectively.

**Table 2:** Comparison of Nausea, Vomiting, Depression, Anxiety and Stress at Hospitalization for Hyperemesis Gravidarum and at the Third Trimester.

	At Hospitalization n = 121	Third Trimester n = 121	P value	Difference (95% Confidence Interval)
Nausea*	121 (100%)	19 (15.7%)	P<0.001	84.3% (76.2%–89.8%)
Vomiting†	121 (100%)	12 (9.9%)	P<0.001	90.1% (82.8%–94.2%)
Depression‡	23 (19.0%)	5 (4.1%)	P<0.001	14.9% (7.2%–23.0%)
Anxiety§	83 (68.6%)	23 (19.0%)	P<0.001	49.6% (38.6%–58.7%)
Stress∥	26 21.5%	4 (3.3%)	P<0.001	18.2% (10.4%–26.4%)

Data expressed as number (%). Analyses were by 2-sided.McNemar's test.

*At least one day of nausea in the last week

† At least one day of vomiting in the last week

‡ A calculated score of at least 10 on the summated (then doubled) scores of the depression component of the 21-stem Depression, Anxiety and Stress Scales

§ A calculated score of at least 8 on the summated (then doubled) scores of the anxiety component of the 21-stem Depression, Anxiety and Stress Scales

∥ A calculated score of at least 15 on the summated (then doubled) scores of the stress component of the 21-stem Depression, Anxiety and Stress Scales

In Table 3, we compared the characteristics of the HG cohort and that of controls recruited in their third trimester (DASS-21 assessment performed at a mean±standard deviation gestational age of 30.5±1.6 weeks). Compared to controls, the HG cohort was significantly (P<0.05) younger, more likely to have had HG in a previous pregnancy and be of Malay ethnicity and less likely to have had a tertiary level education. Adjustment was made for these variables when comparing the risk of nausea, vomiting, depression, anxiety and stress between the HG cohort and controls in the third trimester (displayed in Table 4).

**Table 3:** Characteristics of hyperemesis gravidarum cases at hospitalization and of controls when recruited at 28 weeks.

Characteristics	Hyperemesis Cases n = 121	Controls n = 113	P Value
Age (years)	28.8±4.7	30.7±4.5	P = 0.002
Parity	1 [0–1]	1 [0–1]	P = 0.95
Miscarriage	21 (17.4)	28 (24.9)	P = 0.19
Ethnicity			P<0.001
*Malay*	96 (79.3)	77 (68.1)	
*Indian*	13 (10.7)	12 (10.6)	
*Chinese*	4 (3.3)	24 (21.2)	
*Others*	8 (6.6)	0(0.0)	
Previous hyperemesis gravidarum	31 (25.6)	3 (2.7)	P<0.001
Planned pregnancy	64 (52.9)	48 (42.5)	P = 0.12
Married	118 (97.5)	113 (100)	P = 0.25
Local family support	99 (81.6)	90 (79.6)	P = 0.74
Low income*	53 (43.8)	53 (46.9)	P = 0.69
Housing			P = 0.43
*Owned*	45 (37.2)	50 (44.2)	
*Rented*	67 (55.4)	53 (46.9)	
*Living with extended family*	9 (7.4)	10 (8.8)	
Tertiary Education	70 (57.9)	84 (74.3)	P = 0.01
Paid employment	103 (85.1)	95 (84.1)	P = 0.86
Regular exercise	24 (19.8)	31 (27.4)	P = 0.22

Data presented as mean ± standard deviation, median [interquartile range] and number (%). Bivariate analyses are with Student t test for continuous data set, Mann Whitney U test for ordinal or non-parametric data, Fisher Exact test for 2×2 categorical dataset and Chi Square test for larger categorical dataset. All tests are 2-sided.

*Month income of less than RM3000 (approximately US$950)

**Table 4:** Nausea, Vomiting, Depression, Anxiety and Stress in the Third Trimester in Women Previously Hospitalised with Hyperemesis Gravidarum Compared to Controls.

	Hyperemesis Gravidarum n = 121	Controls n = 113	P value	Relative Risk 95% Confidence Interval	Adjusted P value	Adjusted Odds Ratio (95% Confidence Interval)
Nausea*	19 (15.7)	18 (15.9)	P = 0.99	RR 1.0 95% CI 0.5–2.0	P = 0.43	AOR 1.4 (0.6–3.0)
Vomiting†	12 (9.9)	16 (14.2)	P = 0.42	RR 0.7 95% CI 0.3–1.4	P = 0.49	AOR 0.7 (0.3–1.8)
Depression‡	5 (4.1)	16 (14.2)	P = 0.011	RR 0.3 95% CI 0.1–0.8	P = 0.003	AOR 0.1 (0.03–0.5)
Anxiety§	23 (19.0)	69 (61.1)	P<0.001	RR 0.3 95% CI 0.2–0.5	P<0.001	AOR 0.11 (0.05–0.23)
Stress∥	4 (3.3)	23 (20.4)	P<0.001	RR 0.2 95% CI 0.1–0.5	P<0.001	AOR 0.08 (0.02–0.33)

Data expressed as number (%). Analyses are by Fisher Exact test. All tests are 2-sided. Adjustment made for maternal age, ethnicity, educational attainment and hyperemesis gravidarum in a previous pregnancy as these characteristics are significantly different between the hyperemesis gravidarum and control groups

*At least one day of nausea in the last week

† At least one day of vomiting in the last week

‡ A calculated score of at least 10 on the summated (then doubled) scores of the depression component of the 21-stem Depression, Anxiety and Stress Scales

§ A calculated score of at least 8 on the summated (then doubled) scores of the anxiety component of the 21-stem Depression, Anxiety and Stress Scales

∥ A calculated score of at least 15 on the summated (then doubled) scores of the stress component of the 21-stem Depression, Anxiety and Stress Scales

Table 4 shows the bivariate relative risks and adjusted odds ratios (AOR) of nausea, vomiting, depression, anxiety and stress in the third trimester of the HG cohort compared to controls. There was no difference in nausea and vomiting. The overall nausea and/or vomiting rates were 17.4% compared with 15.9% (95% RR 1.1 95% CI 0.8–1.4; p = 0.86) for HG women against controls in the third trimester. However, depression, anxiety and stress caseness were all far less prevalent (AORs 0.1 [95% CI 0.03–0.5], 0.11 [95% CI 0.05–0.23], and 0.08 [95% CI 0.02–0.33]), respectively in the HG cohort compared to controls.

As HG women of Chinese ethnicity seemed to have a higher rate of depression caseness (Table 1) though numbers were few and the control group has a higher proportion of Chinese women (Table 3), post hoc we performed a sensitivity analysis excluding Chinese women. The results of this sensitivity analysis are not materially changed compared to the original findings as described above.

## Discussion

Currently, the 2-way etiologic relationship between HG and psychological distress remained unresolved. We performed a longitudinal study on a cohort of women with HG assessing the evolution of depression, anxiety and stress from diagnosis of HG into the third pregnancy trimester when in tandem with the typical natural history of HG, full recovery can be anticipated. We also compared the HG cohort against controls (without a history of HG) in the third trimester to address the hypothesis whether psychological distress is driven by the symptoms of HG.

In our study of women hospitalised for HG, using DASS-21 19%, 69% and 21% were classified as having depression, anxiety and stress caseness respectively. No independent risk factor was identified for these components of psychological distress in our cohort. By the third trimester, the rates had fallen to 4%, 19% and 3%, a substantial and significant decrease in tandem with a sharp fall in the symptoms of nausea and vomiting. The HG cohort's adjusted odds ratio for depression, anxiety and stress was only about one-tenth that of controls recruited in the third trimester whilst nausea and vomiting prevalences were similar. The reduction in depression, anxiety and stress was a surprise finding particularly in terms of magnitude; the expectation was that psychological distress in the HG cohort should fall to about the background rate in tandem with the expected fall in the symptoms of nausea and vomiting as HG resolved. The large fall in psychological distress in the HG cohort is not likely to be consistent with psychological distress being a major driver of HG as psychological distress is similar in the first and third trimester of pregnancy [8] and far more supportive of psychological distress being a reaction to the debilitating physical effects of HG, with a very strong rebound in psychological wellbeing after physical recovery from HG. Women with HG also regard HG as being biologically determined.[16] These findings suggest that as perceived by the patients themselves, specific psychological assistance maybe of limited value during acute HG and when offered was typically declined.[7]

A previous report from our centre but using Hospital Anxiety and Depression scale (HADS) had demonstrated depression and anxiety caseness rates at hospitalization for HG of 47.8% and 46.9% respectively: paid employment was an independent risk factor for anxiety and previous miscarriage a “protective” factor against depression caseness.[7] These depression and anxiety rates are quite different from the rates using DASS-21 in our current study of 19% and 69%. In Malaysian women attending a fertility clinic, the anxiety domain of the Malay version of DASS-21 had good correlation with the anxiety domain in HADS but for DASS-21 depressive domain, DASS-21 had modest correlation with its respective domain in HADS.[13] In contrast, we did not identify any independent risk factor for depression, anxiety or stress caseness in the current study. The observed differences between the studies could probably be accounted for by the difference in the performance of DASS-21 and HADS instruments.

When assessed at 15 weeks and again at 20 weeks gestation, depression, anxiety and stress scores have been shown to be higher in 164 nulliparous HG women than in 3259 nulliparous controls with an even greater difference observed in women with severe (defined as requiring hospitalization) HG. That study also reported that elevated stress, depression and limiting response to pregnancy scores occurs secondary to the HG and normalise when the HG improves, although this effect may take weeks to occur. In contrast, more than five weeks following the cessation of vomiting, anxiety scores remain elevated in women with HG.[17] Our data of women hospitalised with HG showed that further differentiation in HG severity using laboratory and clinical parameters did not impact further on the risk of depression, anxiety and stress (Table 1).

The nausea and/or vomiting rates in the third trimester of 15.9% in our control group (which was similar to that in the HG group of 17.4%) may seem high and a potential contributor to psychological distress in controls. We did not exclude women with mild NVP from our control group. A recent meta-analysis of the worldwide literature taking into account data from 59 studies found an average NVP rate of 69.4% with NVP symptoms continuing into the third trimester in 23.5%[9] which would suggest that the 15.9% NVP rate in our controls and 17.4% rate in HG cases were consistent with the global experience.

There were strengths and limitations to our study. Our HG cohort was exclusively of women with the most severe clinical presentation that needed hospitalization. Hospitalization is a useful and pragmatic demarcator of HG from the much milder nausea and vomiting of pregnancy which can affect up to 90% of pregnancies.[18] Our HG cohort sample size was properly powered to observe a drop in depression caseness to the background rate in tandem with expected resolution of HG by the third trimester. The drop-out rate in the study was low and there were few missing data. We presented a hybrid analysis with cohort and case controlled elements which we believed best describe the temporal and case-control correlation between HG and depression, anxiety and stress. However, the Malay language version of DASS-21 has not specifically been validated in HG even though it has been validated against HADS in infertility patients[13]. Longitudinal data starting from prepregnancy to term is required to best define the etiological relationship between psychological distress and HG. This type of data is difficult to obtain as the incidence of HG can be as low as 0.3%, requiring a very large prepregnant sample size for a powered study. We did not take into account factors which might have arisen by the third trimester that might have contributed to depression, anxiety and stress in controls e.g. gestational diabetes, pregnancy induced hypertension, fetal growth restriction. However, HG is not associated with gestational diabetes or hypertension; any positive association that HG might have with fetal growth restriction would tend to move the effect to null instead of a reduction in psychological distress when compared to controls. Also, we took only gestational age into account when selecting our controls which resulted in the control group having a number of characteristics significantly different from the HG cohort. However, we used multivariable logistic regression to adjust for these differences in the eventual analysis. Sensitivity analysis excluding women of Chinese ethnicity also did not materially alter our findings.

### Conclusion

Depression, anxiety and stress in HG are probably in the causal pathway of HG as a response to the deleterious physical effects. The psychological distress appears to be self-limiting in tandem with symptoms of HG. Care in HG should arguably be focused on relieving the symptoms of nausea and vomiting.
